# Current Outlook on Autophagy in Human Leukemia: Foe in Cancer Stem Cells and Drug Resistance, Friend in New Therapeutic Interventions

**DOI:** 10.3390/ijms20030461

**Published:** 2019-01-22

**Authors:** Katharina Rothe, Vanessa Porter, Xiaoyan Jiang

**Affiliations:** 1 Terry Fox Laboratory, BC Cancer Agency, Vancouver, BC V5Z 1L3, Canada; krothe@bccrc.ca; 2 Department of Medical Genetics, University of British Columbia, Vancouver, BC V6H 3N1, Canada; vporter@bcgsc.ca; 3 Genome Sciences Centre, BC Cancer Agency, Vancouver, BC V5Z 1L3, Canada; 4 Department of Medicine, University of British Columbia, Vancouver, BC V5Z 1M9, Canada

**Keywords:** autophagy, hematological malignancies, chronic myeloid leukemia, acute myeloid leukemia, cancer stem cells, leukemia, leukemic stem cells, drug resistance, autophagy inhibitors

## Abstract

Autophagy is an evolutionarily conserved cellular recycling process in cell homeostasis and stress adaptation. It confers protection and promotes survival in response to metabolic/environmental stress, and is upregulated in response to nutrient deprivation, hypoxia, and chemotherapies. Autophagy is also known to sustain malignant cell growth and contributes to cancer stem cell survival when challenged by cytotoxic and/or targeted therapies, a potential mechanism of disease persistence and drug resistance that has gathered momentum. However, different types of human leukemia utilize autophagy in complex, context-specific manners, and the molecular and cellular mechanisms underlying this process involve multiple protein networks that will be discussed in this review. There is mounting preclinical evidence that targeting autophagy can enhance the efficacy of cancer therapies. Chloroquine and other lysosomal inhibitors have spurred initiation of clinical trials and demonstrated that inhibition of autophagy restores chemosensitivity of anticancer drugs, but with limited autophagy-dependent effects. Intriguingly, several autophagy-specific inhibitors, with better therapeutic indexes and lower toxicity, have been developed. Promising preclinical studies with novel combination approaches as well as potential challenges to effectively eradicate drug-resistant cells, particularly cancer stem cells, in human leukemia are also detailed in this review.

## 1. Macroautophagy Is a Cellular Recycling Process and Is Tightly Regulated

Macroautophagy, hereafter autophagy, is a catabolic cell recycling process that degrades cellular components to increase nutrient availability and eliminate toxic waste. This process was coined “autophagy”, meaning “self-eating”, by Christian de Duve at the 1963 Ciba Foundation symposium on lysosomes, to describe the digestion of cellular components [1]. Since the characterization of autophagy by Dr. Yoshinori Ohsumi in 1992, research into its roles in homeostasis, immunity, ageing, cancer, and other diseases have been rapidly progressing [1,2]. Autophagy is especially important for cells that undergo hypoxia, stress, and quiescence, making it a key protective mechanism of stem cells such as hematopoietic stem cells (HSCs) or cancer stem cells, including leukemic stem cells (LSCs). For example, HSCs need to clear harmful oncogenic waste such as reactive oxygen species (ROS) and utilize autophagy for this process [3,4,5]. However, upon oncogenic transformation of HSCs, the same protective attributes of autophagy can become utilized by LSCs to protect them from the hypoxic microenvironment and exposure to therapy [6,7]. Therefore, it is important to understand that autophagy can have multiple, context-dependent roles with therapeutic implications, which will be further explored and explained in this review later on.

Once activated, the autophagy process proceeds by sequestering degradation targets in an independent double-membrane vesicle, followed by fusion with a lysosome for degradation [8,9]. The process is separated into four main parts: induction, nucleation, elongation, and fusion/completion. All the players in this process were first characterized in yeast and then homologous proteins were found in mammalian cells. For the purposes of this review, the mammalian homologues will be described.

**Figure 1:**
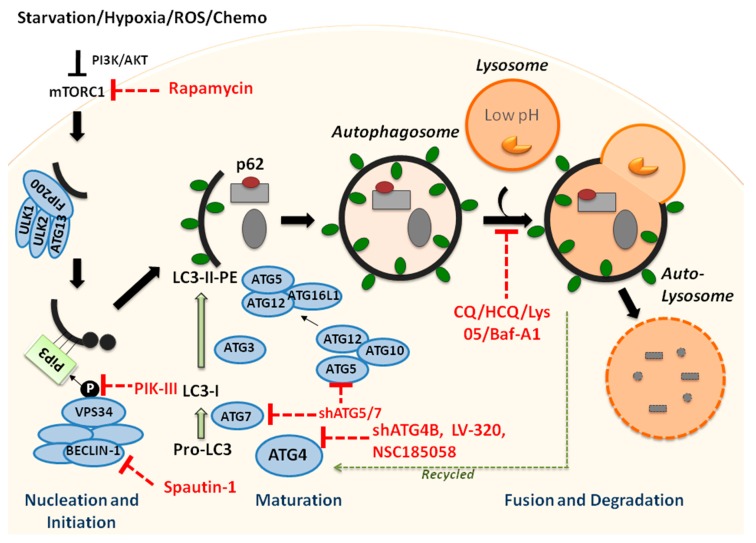
Schematic overview of the autophagy process and genetic and drug targets to inhibit specific aspects of autophagy. Macroautophagy involves four main steps: induction, nucleation, elongation, and fusion/completion. Upon inhibition of mTORC1 by PI3K/AKT, the autophagy process is initiated, for example, when cells undergo starvation. The autophagy process then begins with the formation of a double-membrane structure, the phagophore, which elongates and matures into an autophagosome, sequestering the cytoplasmic cargo. The autophagosome fuses with lysosomes, generating autolysosomes, and the engulfed content is degraded through proteases, and macromolecules are released into the cytosol. Various, indicated ATG proteins and complexes facilitate certain steps in this catabolic pathway. In addition, the conversion of LC3-I to LC3-II, as well as the degradation of p62, together with the enclosed cargo, serve as markers of active autophagy in molecular and immunochemical assays. Chloroquine and other lysosomal compounds, as well as newly developed inhibitors and genetic targets to specifically block the autophagy pathway at certain points, are indicated. ROS = Reactive oxygen species; PI3K = Phosphoinositide3-kinase; AKT = Protein kinase B; mTORC1 = mammalian target of rapamycin complex I; ATG = Autophagy-related gene; ULK = Unc-51-like autophagy activating kinase; FIP200 = FAK family-interacting protein of 200 kDa; PiP3 = Phosphatidylinositol(3,4,5)-trisphosphate; VPS34 = Class III PI3K vacuolar protein sorting 34. Solid black and green arrows indicate activation and direction of autophagy steps; red dotted lines show inhibition; dotted green arrow indicates recycling of LC3-II-PE by ATG4s.

The initiation complex of autophagy is comprised of the ULK1/2-ATG13-FIP200 interacting proteins, which remain stable in the cell regardless of nutrient availability (Figure 1) [10]. Dysregulation of the initiation complex, and pathways upstream of the autophagy initiation complex, can significantly alter cellular autophagy levels, making it important for proper metabolic function. This complex actively initiates autophagy unless it is inhibited through phosphorylation of ULK1/2 and ATG13 by mTORC1, which is up-regulated by high nutrient density in the cell, as well as many other processes [10]. Upon cell starvation or mTORC1 inhibition, the initiation complex is released, and it activates nucleation through phosphorylation of BECN1 within the ATG14-containing class III phosphatidylinositol 3-kinase (PtdIns3K) complex [11]. The PtdIns3K complex localizes at the site of the phagophore generation, which can be the endoplasmic reticulum, the plasma membrane, the golgi apparatus, the mitochondria, or other double membrane structures [12]. This complex contains PIK3C3, PIK3R4, PtdIns3K, and BECN1, with BECN1 being the most crucial for regulation and induction of autophagy [13,14]. VPS34 deposits lipid phosphatidylinositol (PI) on the surface of the phagophore, which is then phosphorylated into phosphatidylinositol-3-phosphate (PI3P) and serves as a docking site for proteins bringing degradation targets into the autophagosome [15]. Several family members of the initiation complexes such as BECN1 and VPS34 are subjected to genetic mutations or altered gene expression in human leukemia, which can cause significant molecular and cellular downstream effects in the autophagy process [7,14,16,17].

Next, the elongation step requires the conjugation of two ubiquitin-like (UBL) proteins to the membrane of the phagophore for it to mature into an autophagosome (Figure 1) [18]. Members of the lipidation cascade during phagophore elongation are subjected to many mutations and dysregulation in human leukemia, and loss of key enzymes in the cascade can cause complete loss of autophagy. The first system forms the ATG12-ATG5-ATG16L1 dimeric complex, which associates with the membrane via attachment of ATG16L1 [18]. The complex is brought together through the irreversible conjugation of the UBL protein ATG12 to ATG5, catalyzed by the E1-like activating enzyme ATG7 and the E2-like conjugating enzyme ATG10 [19,20,21]. ATG16L1 binds to ATG5 and then ATG16L1 dimerizes with another ATG12-ATG5-ATG16L1 complex, followed by attachment to the phagophore membrane [22]. The second UBL conjugation system attaches lipidated mammalian ATG8 homologues to the autophagosome membrane; these homologues are divided up into the MAP1-LC3 (LC3) and GABARAP subfamilies, with the LC3 system being the most understood [15,23]. The translated LC3 protein is first cleaved by ATG4 protein homologues (most abundantly ATG4B) into LC3-I, which has an exposed C-terminus glycine residue necessary for subsequent conjugation [24]. The E1-like activating enzyme ATG7 then transfers LC3-I to the E2-like conjugating enzyme ATG3 [25]. The ATG12-ATG5-ATGL1 complex recruits lipid phosphatidyethanolamine (PE) from lipid membranes and catalyzes the lipidation of LC3-I to PE and the attachment of PE to the membrane to form LC3-II [26]. Once the autophagosome is fully formed, ATG4 cleaves off the PE domain from LC3-II to release LC3-I back into the cytosol to be reused in subsequent rounds of the process [27]. Members within this cascade such as ATG4B, ATG7, and ATG5 not only show dysregulation in leukemia, but can also be effectively targeted to halt the autophagy process, thus, making interruption of autophagosome maturation an attractive area of study [6,28,29].

In the final step of autophagy, the autophagosome must close off from the cytosol and fuse with the lysosome to degrade its contents (Figure 1). This is the most commonly targeted step in the autophagy pathway because of the well-characterized lysosomal inhibitors chloroquine (CQ) and hydrochloroquine (HCQ) [30]. The completed autophagosome is trafficked through the cell to the lysosomes by microtubules, and then fuses with the lysosome to form an autophagolysosome [31]. The acidic hydrolases within the lysosome degrade the macromolecules engulfed by the autophagosome into their smaller components, such as amino acids, nucleotides, fatty acids, or carbohydrates [32]. These molecules are released back into the cytosol to be recycled in metabolic processes.

Autophagy is a tightly-regulated catabolic response to numerous types of cellular stresses such as nutrient starvation, hypoxia, growth factor deprivation, endoplasmic reticulum (ER) stress, various metabolic challenges, and importantly, cancer therapy drugs [33]. Various proteins and pathways are responsible for sensing the levels of these stressors within the cell and then inhibiting or inducing the autophagic response, and many of them are dysregulated in human hematological malignancies. The carbon and nitrogen content of the cell is detected by cAMP-dependent protein kinase A (PKA) and mTOR pathways, and these two mediators block autophagy in nutrient-rich conditions [34,35]. The energy-sensing kinase AMP-activated protein kinase (AMPK) up-regulates autophagy through ULK1 phosphorylation during low-energy conditions, as determined by the AMP/ATP ratio [11]. ER stress activates autophagy when the cytosolic Ca^2+^ concentrations increase and the calmodulin-dependent protein kinase kinase 2 (CAMKK2) activates AMPK, or when unfolded proteins in the ER accumulate and induce autophagy [36]. Hypoxia also positively regulates autophagy through inhibition of mTORC1 by reactive oxygen species (ROS) [37]. Several other pathways that become activated by extracellular signals, such as growth factors and insulin, are down-regulated in the absence of ROS and result in an upregulation of autophagy [38]. This adaptive and sensitive response of a cell to changing conditions and nutrient availability promotes homeostasis and cell viability and is important to the integrity of hematopoietic stem cells.

## 2. Autophagy Is Critical in the Maintenance of Hematopoietic Stem Cells

HSCs are a rare population that ensure life-long hematopoiesis, and as such must be carefully maintained and protected to counteract damage and accumulation of mutations that could initiate or promote leukemia development [39,40,41]. Several studies have shown that autophagy plays key roles in the maintenance and metabolism of murine HSCs. Conditional, hematopoietic-specific deletions of the essential autophagy genes *Atg7*, *Atg5*, or the ULK1-interacting partner *Fip200* in a mouse model diminished normal HSC activities, promoted a pre-leukemic phenotype, and consequently impaired survival of these mice [3,29,42]. Moreover, Mortensen et al. demonstrated that LSK cells (lin^−^Scal^+^cKit^+^, mouse stem/progenitor cells) from *Atg7* knockout mice displayed an accumulation of mitochondria, mitochondrial superoxide, and DNA damage, with increased cell proliferation rates [29]. Warr et al. showed later that mouse HSCs quickly induce autophagy upon metabolic challenges and that this adaptive response is driven by the pioneer transcription factor *FOXO3A* [4]. Interestingly, a proportion of aged murine HSCs have, similar to young HSCs, high basal autophagy levels with robust long-term regenerative potential, while most HSCs in aged mice, or *Atg12* knockout HSCs, exhibit overactive mitochondrial metabolism, loss of quiescence, and expansion of the myeloid compartment [5]. Together, these data indicate that the functions of HSCs, at least in part, depend on proficient autophagy and that perturbations in autophagy in these cells can pave the path for the initiation and development of hematological malignancies.

## 3. Autophagy Plays Context-Dependent Roles in Leukemia Initiation, Progression, and Drug Resistance

Leukemia is often referred to as a clonal stem cell disorder where self-renewing LSCs have been described to initiate tumor formation and later cause chemotherapy resistance or failure and disease relapse [43,44,45,46]. LSCs can either originate from transformed HSCs or their more differentiated and mutated progeny, depending on the type of leukemia, disease stage, and other contributing factors [47,48,49,50]. Intriguingly, several studies have shown that LSCs and leukemic blasts can utilize autophagy to respond to the specific energetic demands during accelerated cell proliferation and to counteract chemotherapeutic stress, to ensure their survival. For example, in chronic myeloid leukemia (CML), we and others demonstrated that patient-derived LSCs possess high levels of basal autophagy gene expression compared to more mature cells or their normal counterparts, and that targeting autophagy by genetic or pharmacological inhibition resulted in reduced leukemic cell viability and enhanced sensitivity to standard chemotherapy [6,7,51]. In contrast, studies in acute myeloid leukemia (AML) suggest a different function for autophagy, since autophagy seems often to be reduced in human AML blasts and loss of key autophagy genes leads to leukemia initiation and progression in mouse models [42,52,53]. Interestingly, in either case, autophagy can have cytoprotective roles that can be utilized to enhance chemotherapeutic agent sensitivity in leukemic cells [6,54]. These seemingly paradoxical roles for autophagy highlight its complexity and context-specific functions, and hence, will be discussed in more detail in the context of each leukemia separately.

### 3.1. The Molecular and Functional Roles of Autophagy in CML

CML is a multi-lineage myeloproliferative neoplasm that originates from HSCs and is characterized by uncontrolled proliferation of hematopoietic cells, particularly an excessive number of granulocytes in the peripheral blood. More than 95% of patients harbor a characteristic reciprocal chromosomal translocation product, called *BCR-ABL1*, that originates in a HSC and leads to overexpression of its protein (p210^BCR-ABL^), with constitutively activated tyrosine kinase activity, giving rise to and sustaining the CML clone [55,56,57]. The identification of BCR-ABL1 as the major driver in the pathogenesis of CML led to the development of targeted therapies with ABL1 tyrosine kinase inhibitors (TKIs) such as Imatinib, Dasatinib, and Ponatinib [58,59]. Treatments with TKIs in the initial chronic phase of CML have been shown to induce remarkable haematological and cytogenetic responses, however, treatments of advanced stage patients or individuals with ABL1 kinase domain mutations are much more challenging and most importantly, none of these monotherapies are curative [60,61,62]. Strong evidence indicates that LSCs, including quiescent LSCs, are insensitive to TKIs and constitute a critical source of leukemia recurrence and a significant reservoir for the emergence of TKI-resistant sub-clones, necessitating lifelong treatment for most patients [57,63,64,65,66,67,68,69,70]. Interestingly, it has been reported that CML stem cells are not strictly dependent on the tyrosine kinase activity of BCR-ABL1 for survival and that they may be effectively protected by the bone marrow microenvironment and/or exploit signaling pathways such as autophagy [51,71,72,73].

The connection between autophagy and CML was first documented by an increased autophagic response following treatment with imatinib mesylate (IM) or other non-targeting drugs in CML cell lines and primary patient samples [51,74]. This response was attributed to ROS generation, ER stress, and a dose-dependent increase in gene and protein expression of key autophagy players [51,75,76]. The increase in autophagy induces CML cells to become senescent rather than apoptotic, causing them to be more elusive to treatment [77]. Thus, the regulation of autophagy in CML has been largely explored in the context of BCR-ABL1, but may be influenced by different signaling pathways. For example, the mammalian target of rapamycin (mTOR) in the TORC1 signaling pathway is a well-known negative regulator of autophagy, and BCR-ABL1 has been shown to suppress autophagy via the PI3K/AKT/FOXO4/ATF-5 pathway by stimulating transcription of mTOR [78,79,80]. On the other hand, it has been shown that autophagy is induced by BCR-ABL1 via the rapamycin-insensitive mTORC2 signaling complex and helps CML cells to recover from TKI treatment [74,81]. The kinase mTOR can be part of two distinct complexes, TORC1 and TORC2 [80]. While the TORC1 complex and its negative regulation on autophagy has been extensively studied, much less is known about the TORC2 complex that stimulates and mediates autophagy during amino acid starvation [80]. TORC2 promotes autophagy via its downstream target Ypk1, which in turn inhibits calcineurin, allowing activation of the eIF2α kinase Gcn2 and phosphorylation of 4E-BP1 T37/46 and S65 to activate autophagy [80].

Key enzymes in the LC3 conjugation cascade, such as ATG7 and ATG4B, have both been implicated as critical for CD34^+^ CML cell survival, while genetic inhibition of ATG4B or ATG7 was able to increase apoptosis of CML stem and progenitor cells and sensitize them to TKIs [6,28]. Interestingly, knockdown of ATG7 also led to metabolic reprogramming that resulted in increased oxidative phosphorylation and mitochondrial ROS accumulation and caused primary CML cells to differentiate into erythroid cells [28]. Mechanistically, a recent study demonstrated that ULK1 could inhibit ATG4B activity and LC3 processing by phosphorylating ATG4B on serine 316, resulting in inhibition of its catalytic activity both in vitro and in vivo [82]. Notably, this activity seems to be regulated by PP2A-mediated dephosphorylation. MST4, a member of the mammalian sterile20-like (STE) serine/threonine kinase (STK) family, was found to stimulate autophagy by activating ATG4B through phosphorylation of ATG4B S383, which stimulates ATG4B to act on LC3, thereby increasing autophagic flux [83]. Of even more interest, inhibiting ATG4B can enhance the anti-tumor effects of radiotherapy in a solid tumor model. Our group investigated ROS-mediated autophagy in the context of protein–protein interactions between BCR-ABL1, the scaffold protein AHI-1, and the cytoskeletal modulator DNM2 [75]. Here, the identified AHI-1-BCR-ABL-DNM2 complex was shown to regulate endocytosis and ROS generation, both of which lead to autophagy induction in CML, but knockdown of DNM2 disrupted these activities and reduced autophagy induction in primitive leukemic cells [75]. Ianniciello et al. also demonstrated that proficient autophagy is required for CD34^+^ CML cells to proliferate when they transition from hypoxic to normal oxygen conditions, which mimics the leukemic commitment as the cells migrate away from the hypoxic bone marrow niche [84]. Together, these findings reinforce the concept that autophagy is a critical pro-survival pathway in CML and contributes to drug resistance and disease progression. The recognition of the innate and acquired mechanisms that make CML stem cells resistant to TKIs has thus prompted considerable interest in developing new treatment strategies, including targeting autophagy.

### 3.2. The Molecular and Functional Roles of Autophagy in AML

Unlike CML, AML is a very heterogeneous disease and represents much more context-dependent complexity regarding the role of autophagy in disease initiation and progression. Acute leukemias are characterized by the rapid accumulation of clonal, immature myeloid blasts, with incomplete differentiation, in the patients’ bone marrow and peripheral blood, and are often accompanied by multi-lineage cytopenias [85,86]. Although major progress has been made in identifying different molecular and genetic subgroups, AML therapies and long-term patient outcomes have not improved significantly over the past 40 years [87,88]. One of the foremost hurdles to a cure is thought to be the presence of LSCs or leukemia-initiating cells that, in many patients, constitute a small subpopulation of self-renewing cells at the top of a hierarchical organization with resistance to chemotherapy, causing treatment failure and relapse [89,90,91,92]. Immunodeficient mouse models show that these leukemic cells can produce AML upon xenotransplantation, including in secondary and tertiary recipients, and generate non-LSC committed progenitors unable to be serially transplanted, all features of functionally true LSCs [90,91,93,94,95]. Initial immunophenotypic analyses demonstrated that the majority of AML patient cells express the surface antigen CD34^+^, with LSCs mostly residing in the CD34^+^CD38^−^ subfraction [91,96,97]. However, more recent studies revealed that many patients harbor, at minimum, two distinct LSC populations that coexist in the CD34^+^CD38^−^ (Lymphoid-primed multipotent progenitor (LMPP)- or multipotent progenitor (MPP)-like LSCs) and in the CD34^+^CD38^+^ (granulocyte-macrophage progenitor (GMP)-like LSCs) subfractions or less often in the CD34^−^ subfraction, with the former, more immature CD34^+^CD38^−^ subpopulation containing a higher LSC frequency in comparative xenotransplantation experiments [98,99,100,101]. Moreover, some AML patients lack expression of CD34, the CD34^−^ AML cells, and here, LSCs can mostly be detected in the CD34^−^ compartment, with very few LSCs being present in the much smaller CD34^+^ fraction [99,100,102,103], highlighting the heterogeneity of AML and the complexity of studying and understanding the disease.

AML patients usually harbor genetic abnormalities that often originate from chromosomal translocations and rearrangements, such as t(8;21), t(15;17), inv(16), t(6;9), t(9;11), or t(11;19), and characterize, in some cases, a particular prognostic leukemic subtype [104,105,106,107]. Recent molecular studies demonstrated that additional mutations in receptor kinases, key signaling mediators, proto-oncogenes, or epigenetic enzymes, for example FLT3-ITD, TP53, c-KIT, or IDH1/2 mutations, often determine the course and severity of the disease [108,109,110,111,112,113]. Interestingly, sequencing and in silico studies have shown that a high frequency of AML patients carry often heterozygous deletions, missense mutations, or copy number changes of key autophagy genes, particularly AML patients with complex karyotypes [42,114,115]. For example, heterozygous chromosomal loss of 5q, 16q, or 17p correlate with the encoded regions for the autophagy genes *ATG10* and *ATG12*, *GABARAPL2* and *MAP1LC3B*, or *GABARAP*, respectively [42]. In line with these observations, Watson et al. also demonstrated that human AML blasts exhibit low expression of several autophagy genes, including *ATG10*, *ATG5*, *ATG7*, *BECN1*, *GABARAP*, *GABARAPL1/2*, and *MAP1LC3B*, decreased autophagic flux, and high ROS levels [42]. Furthermore, this study showed that heterozygous loss of *Atg5* or *Atg7* in a MLL-ENL AML mouse model led to more aggressive leukemia progression, suggesting a tumor-suppressive role for autophagy [42]. Similarly, Jin et al. confirmed that Ficoll-enriched leukemic blasts from AML patients express significantly lower transcript levels of *ULK1*, *FIP200*, *ATG14*, *ATG5*, *ATG7*, *ATG3*, *ATG4B*, and *ATG4D* compared to granulocytes from healthy donors [52]. In addition, Rudat et al. determined, in a large RNAi screen for “rearranged during transfection” receptor tyrosine kinase (RET) effectors, that mTORC1-mediated suppression of autophagy can stabilize mutant FLT3 in AML, while an increase in autophagy was achieved through RET inhibition and led to FLT3 depletion [53]. In contrast, Heydt et al. showed that FLT3-ITD increases autophagy in AML cell lines and patient cells via ATF4 and that inhibition of autophagy or ATF4 abolishes FLT3 inhibitor resistance [116]. Moreover, a recent investigation by Folkerts et al. revealed that various leukemic cell lines, and purified CD34^+^ cells from AML patients, exhibit inconsistent levels of basal autophagic flux, with particularly high levels in immature ROS^low^ LSC blasts and adverse AML risk groups, such as those with TP53 mutations [117]. In this study, knockdown of ATG5 in primary AML cells resulted in impaired engraftment of human cells in immunodeficient NSG mice, an observation that is in contrast to previous work and would rather suggest a tumor-promoting role for autophagy in this context [117]. Interestingly, other reports showed that ATG5 or ATG7 are required for the efficient initiation of AML in the context of MLL-AF9, the most common alteration found in infant AML, with poor prognosis, but that autophagy is no longer needed for the maintenance of established AML or LSC functions in secondary xenotransplantation experiments [54,118,119,120]. However, in a different murine MLL-ENL AML model, knockout of *Atg5* or *Atg7* decreased the number of functional LSCs, increased activation of mitochondria and ROS levels in these cells, and extended survival of leukemic mice [121]. Together, these representative, variable data suggest a highly complex, context-dependent role for autophagy in leukemic transformation vs. maintenance and LSC properties in AML.

Notably, and similar to CML, many studies in AML have shown another consistently critical role for autophagy with treatment implications: pro-survival protection of leukemic cells upon chemotherapy. For instance, it has been demonstrated that treatment of AML cells with cytarabine (AraC), anthracyclines, or sorafenib activates and increases autophagic flux in these cells, including LSCs, allowing them to resist chemotherapy [54,121,122]. However, in these cases, targeting of autophagy by genetic or pharmacological means, combined with chemotherapy, seems to hold great promise in developing more effective treatment strategies, as discussed later. 

### 3.3. The Molecular and Functional Roles of Autophagy in Lymphocytic Leukemia

Lymphocytic leukemias arise in the bone marrow from a lymphoid progenitor cell, or in the case of chronic lymphocytic leukemia (CLL) from marginal zone B-cells, and take on either a blast-like T-cell or B-cell phenotype [123,124]. Acute lymphocytic leukemia (ALL) is the most common pediatric cancer and, much like AML, ALL is widely diverse in its presentation and driver mutations and progresses quickly if left untreated. CLL—the most common adult leukemia—is more indolent and differentiated than ALL but is also genetically diverse [123]. Within ALL cases, the most common phenotype is the B-cell-like leukemia (B-ALL), and CLL only presents as B-cell-like. Targeted treatments for lymphocytic leukemia are still limited due to the molecular heterogeneity, but clinical advances have greatly improved the prognosis of these diseases with non-targeted chemotherapies [123,124,125,126]. However, subsets of patients acquire therapeutic resistance through unknown mechanisms, and both ALL and CLL have identified autophagy as a potential resistance pathway.

An autophagic treatment response has been identified in lymphoblastic leukemia, specifically after metabolic chemotherapeutics used to target ALL. Standard treatment of B-ALL is the administration of synthetic glucocorticoids (GC) to slow down cellular glucose intake and induce cell death [127]. Following GC treatment in B-ALL, there is a reduction in glucose and glutamine cellular intake but unexpectedly an increase of glutamine synthesis [128]. Dyczynski et al. showed that in the absence of normal nutrient intake, there is increased autophagic flux to catabolically make up for the lost nutrients; a byproduct of this catabolism, ammonia, is then utilized by glutamate–ammonia ligase to synthesize glutamine intracellularly, allowing the cells to provide their own glucose supply [128]. B-ALL patients that were GC-resistant also had 36 differentially expressed autophagy genes (26 upregulated and 10 downregulated) compared to GC-sensitive patients, suggesting the autophagic GC response is important for treatment-mediated apoptosis [129]. ALL treatment with l-asparaginase (l-asp) also targets metabolism by hydrolyzing glutamine and asparagine to glutamic acid and aspartic acid, respectively, thus reducing aerobic metabolism [126]. Upon l-asp treatment, ALL cells induce autophagy to compensate for the metabolic stress, and inhibition of autophagy using CQ induces treatment sensitivity in vitro and in vivo [130].

There are genetic connections between lymphoblastic leukemia driver mutations and autophagy. B-ALL patients with the ETV6-RUX1 translocation have significantly increased expression of the autophagy initiating lipidase VPS34 compared to normal hematopoietic stem/progenitor cells, and the presence of the ETV6-RUNX1 fusion protein was found to have a causal relationship to VPS34 overexpression through epigenetic regulation [131]. In CLL, a positive prognostic factor is the deletion of 13q14, containing microRNA-15 and -16, both of which target anti-apoptotic B-cell CLL/lymphoma 2 (BCL-2) leading to BCL-2 upregulation in CLL [132]. BECN1 is negatively regulated by the binding of BCL-2, and therefore BCL-2 upregulation suppresses BECN1 activity and inhibits autophagy [133]. Another positive prognostic factor of CLL is the expression of SLAMF1, which is involved in autophagosome recruitment and autophagic flux activation [134]. These positive CLL prognostic markers (13q14 deletion and stable SLAMF1 expression) have opposing downstream mechanistic effects on autophagy, yet both positively affect CLL prognosis, showcasing once more how molecular heterogeneity can change the role of autophagy.

## 4. Targeting Autophagy Is Critical to Eradicate Drug-Resistant and Cancer Stem Cells in Human Leukemia

Overcoming drug resistance and eliminating cancer stem cells remain major challenges in the treatment of human leukemia and other cancers. According to the cancer stem cell model, it will be necessary to eliminate LSCs to achieve a long-term cure for human leukemia. LSCs are at the root of the disease and evidence indicates that these are the cells that endure standard chemotherapy and expand upon relapse [57,135]. Notably, many studies have shown that autophagy plays a key role in the cytoprotection of leukemic cells, including LSCs, and hence, autophagy has been intensively investigated as a promising, novel target, with most approaches focusing on inhibition of autophagy with combination treatments (Figure 1 and Table 1). In this context, noteworthy is a phase II clinical trial that combines hydroxychloroquine (HCQ) with Imatinib in CML (NCT01227135, CHOICES: CHlOroquine and Imatinib Combination to Eliminate Stem cells). However, three other phase I/II clinical trials strongly suggest that specificity and potency issues with HCQ will require more selective and potent autophagy inhibitors to achieve optimal responses in cancer patients [30,136,137,138]. Baquero et al. have lately explored second-generation autophagy inhibitors in CML models [7]. By combining Lys05, an analogue of CQ, or PIK-III, a selective VPS34 inhibitor, with the TKI nilotinib, the group was able to demonstrate more effective targeting of viable human LSCs in vitro and in vivo compared to single agents [7]. Recently, specific ATG4B inhibitors were developed for pre-clinical proof-of-concept studies [139,140]. LV-320, a styrylquinoline, was identified by in silico screening and high content cell-based screening. It can inhibit autophagic flux, shows excellent tolerability, and has a good pharmacokinetics (PK) profile [140]. Treatment with LV-320 of several drug-resistant and mutated CML and aggressive BCR-ABL1^+^ B-ALL cell lines and TKI-insensitive CML patient LSCs effectively inhibited cell growth and these effects could be enhanced when combined with TKIs [141,142]. Although the biological effects of the ATG4B antagonist NSC185058 have not been described in hematological malignancies, it was recently reported that it can suppress the activation and lipidation of LC3B and the degradation of p62/SQSTM1, which result in significantly reduced cell viability and xenograft growth in vitro and in vivo when combined with CQ in a glioblastoma model [83]. Modulators of autophagy regulation have also been investigated as treatment options. Indirect targeting of the PI3K/AKT pathway with the potent and specific autophagy inhibitor Spautin-1, which can induce an increase in proteasomal degradation of class III PI3K kinase, enhanced IM-mediated apoptosis by reducing the expression of the anti-apoptotic proteins MCL-1 and BCL-2, and down-regulation of key autophagy genes such as BECN1 in CML [143].

Interestingly, autophagy-mediated resistance can also be exploited without the use of TKIs in CML cells. By inhibiting hedgehog signaling, a critical pathway in the persistence of LSC, autophagy was shown to be induced, but targeting of both hedgehog signaling and autophagy effectively eradicated BCR-ABL1^+^ leukemic cells without the need for TKIs [146]. Similarly, Lu et al. revealed that resistant CML cells upregulate autophagy upon treatment with Tigecycline, however, concurrent inhibition of autophagy and mitochondrial translation decreased viability of drug-resistant cells [144]. This observation was further supported by an elegant study demonstrating that targeting mitochondrial oxidative phosphorylation, with a combination treatment of Tigecycline and IM, selectively eliminated CML LSCs in vitro and in a xenotransplantation CML model [154]. In another study, Mitchell et al. demonstrated that upon prevention of mTOR signaling with the dual PI3K/mTOR inhibitors PI-103 or NVP-BEZ235, autophagy became elevated in CML and ponatinib-resistant leukemic cells and in turn effectively sensitized these cells to HCQ treatment [145]. Targeting of mTOR signaling has also been investigated in AML, Ph^+^ALL, and T-ALL [120,147,155,156,157], and has led to a phase I clinical trial with NVP-BEZ235 for dose-escalations and relapsed or refractory AML (NCT01756118).

Similar to CML, numerous studies in AML have focused on inhibiting autophagy to counteract its cytoprotective role upon first-line chemotherapy. Treatment of AML cell lines and primary patient cells as well as LSCs with AraC or cytarabine has been shown to induce autophagy in these cells [54,119,121,122]. However, by combining chemotherapy with ATG7 depletion or CQ/HCQ/Bafilomycin A1 treatment to block autophagy, the therapeutic effects of AraC or cytarabine could be enhanced and cell death promoted, even in drug-resistant cells and the LSC population [54,119,121,122,147,148], suggesting that the autophagy process is critically involved in treatment response/resistance of AML cells. Other agents that have been shown to stimulate autophagy in AML cells and that are currently being investigated in combination approaches, with promising results, include Statins, S100A8, and Arginase [16,17,149,150,151,152,153,158]. Statins act on a key enzymatic step in cholesterol biosynthesis, thereby limiting the availability of cholesterol in cells and promoting the induction of autophagy [149,158]. S100A8 interacts with BECN1 to initiate autophagy and is elevated in resistant AML cells, however, depleting S100A8 sensitizes leukemic cells to chemotherapy [16,17]. Treatment with human, recombinant Arginase, an enzyme that depletes arginine, has been shown to target AML blasts due to their dependency on extracellular arginine, but also increases autophagy in these cells, warranting a combination strategy with 3-methyladenine or CQ to effectively target leukemic cells [150,151,152]. While these approaches seem promising for improved therapeutic interventions upon induced cytoprotective or enhanced basal autophagy in most leukemic cells, specific attention to the other possible roles of autophagy in certain types of leukemia is also needed. For example, in some AML cases, autophagy has been shown to be critical for degradation of oncogenic drivers, such as mutant FLT3, and during drug-induced myeloid or neutrophil differentiation therapies, suggesting that autophagy initiation is beneficial in certain contexts [52,53,159]. Considering these circumstances, additional studies are needed to shed light on the complex roles and mechanistic details of autophagy in the regulation of leukemia initiation and drug response/resistance, in hopes of identifying the best treatment options for leukemia.

## 5. Conclusions

Autophagy is not only fundamental in the maintenance and protection of normal HSCs and progenitors, but also plays critical, albeit complex, roles in the initiation and progression of hematological malignancies and LSC survival. Hence, it seems to be a double-edged sword; while HSCs depend on autophagy to finely tune their need for building blocks under changing nutrient demands and to clear damaged organelles, such as mitochondria, to counteract possible damage by high ROS levels, resistant LSCs in CML express high basal levels of key autophagy genes combined with elevated autophagic flux, and inhibition of autophagy in LSCs decreases their clonal proliferation capacities and sensitizes them to standard targeted therapies with TKIs. Furthermore, the complex and context-dependent functions of autophagy are evident in AML, where both leukemic transformation and leukemia maintenance are highly influenced by the autophagic pathway. Disruption of functional autophagy can lead to efficient initiation and more aggressive disease progressions, for instance in MLL-mutated AML cells, but the autophagy process may also stabilize oncogenic drivers such as FLT3-ITD, which is elevated in poor risk primary AML patient cells and most importantly, ensures survival of leukemic blasts and LSCs upon chemotherapy. Due to the numerous interesting observations that autophagy has a critical cytoprotective role, various approaches have been investigated in model systems, including AML, combining autophagy inhibition with standard chemotherapies, with promising results. However, as prior work has demonstrated, the inter- and possibly also intra-mutational heterogeneity of AML patients and their dominating disease clones will require further detailed studies to translate these findings into safe clinical options in the future. In addition, more specific and highly effective autophagy inhibitors are being developed and multiple investigations are underway, highlighting the importance of autophagy and its potential in improved treatment strategies in hamatological malignancies, especially for patients at high risk of drug resistance and disease progression.

## Figures and Tables

**Table 1:** Autophagy combination treatment strategies in leukemia. CLL = Chronic lymphocytic leukemia; CML = Chronic myeloid leukemia; AML = Acute myeloid leukemia; ALL = Acute lymphoblastic leukemia.

Autophagy Modifier	Combination Strategy	Leukemia Types	Context	Ref
HCQ, Lys05, PIK-III (VPS34), Spautin-1, *shATG4B*, LV-320, NSC185058	+TKIs	CML	In vitro and in vivo	[6,7,83,142,143]
CQ	+l-asp	ALL	In vitro and in vivo	[130]
CQ, *siATG5*	+Tigecycline	CML	In vitro	[144]
HCQ	+NVP-BEZ235	CML	In vitro and in vivo	[145]
HCQ, *shATG7*	+Vismodegib	CML	In vitro	[146]
*atg7^−/−^*	+AraC	Murine AML	In vitro and in vivo	[121]
CQ, Bafilomycin A1, *siMAP1LC3*, *siSQSTM1*	+Cytarabine	AML	In vitro	[147]
CQ, 3-MA, *shATG7*	+AraC	AML	In vitro and in vivo	[54,148]
Bafilomycin A1, *siBECN1*	+Statins	CLL, CML, AML, ALL	In vitro	[149]
−	shS100A8+adriamycin or vincristine	CML, AML, ALL	In vitro	[17]
CQ, 3-MA	+rhArginase	AML, ALL	In vitro	[150,151]
−	rhArginase+AraC or dexamethasone	CML, AML, ALL	In vitro and in vivo	[152,153]
